# Phonological loop affects children’s interpretations of explicit but not ambiguous questions: Research on links between working memory and referent assignment

**DOI:** 10.1371/journal.pone.0187368

**Published:** 2017-10-31

**Authors:** Xianwei Meng, Taro Murakami, Kazuhide Hashiya

**Affiliations:** 1 Graduate School of Human-Environment Studies, Kyushu University, Fukuoka City, Fukuoka Prefecture, Japan; 2 Japan Society for the Promotion of Science, Tokyo, Japan; 3 Department of Education and Psychology, Faculty of Humanities, Kyushu Women’s University, City of Kitakyushu, Fukuoka Prefecture, Japan; 4 Faculty of Human-Environment Studies, Kyushu University, Fukuoka City, Fukuoka Prefecture, Japan; University of Akron, UNITED STATES

## Abstract

Understanding the referent of other’s utterance by referring the contextual information helps in smooth communication. Although this pragmatic referential process can be observed even in infants, its underlying mechanism and relative abilities remain unclear. This study aimed to comprehend the background of the referential process by investigating whether the phonological loop affected the referent assignment. A total of 76 children (43 girls) aged 3–5 years participated in a reference assignment task in which an experimenter asked them to answer explicit (e.g., “What color is this?”) and ambiguous (e.g., “What about this?”) questions about colorful objects. The phonological loop capacity was measured by using the forward digit span task in which children were required to repeat the numbers as an experimenter uttered them. The results showed that the scores of the forward digit span task positively predicted correct response to explicit questions and part of the ambiguous questions. That is, the phonological loop capacity did not have effects on referent assignment in response to ambiguous questions that were asked after a topic shift of the explicit questions and thus required a backward reference to the preceding explicit questions to detect the intent of the current ambiguous questions. These results suggest that although the phonological loop capacity could overtly enhance the storage of verbal information, it does not seem to directly contribute to the pragmatic referential process, which might require further social cognitive processes.

## Introduction

Appropriately interpreting the referent of others’ communicative expressions is essential for smooth and efficient communication [1–2]. Without each utterance carrying every detail and even when its semantic meaning is ambiguous, we routinely infer speakers’ intended meanings by assuming that utterances convey only relevant information [3–4]. Let us imagine a situation in which someone is talking about a female person using the pronoun “she” to refer to the female. Note that although "she" is an arbitrary reference to female individuals, we would not find it hard to understand the referent because the context in which the person is talking indicates that the referent is a specific individual. However, once the talker has mentioned the topic of a second female person, we may interpret the referent of “she” as referring to the person who was mentioned immediately before it. This flexible pragmatic reference assignment process concerns drawing reasonable or appropriate context-independent meaning of an arbitrary utterance, occurs when the referent has appeared at an earlier point in the sequence, and requires interpretation through the preceding context on the basis of the common experience between the participants of communication [5–6]. Studies on preverbal infant communication have suggested that our pragmatic referential tendency seems to be observed from the second year of life, based on the evidence that infants interpret others’ utterances based on tracking their common experiences [7–11]. For example, when infants were asked to retrieve one ball from two balls using the ambiguous utterance of “Where’s the ball?”, 14-to-20-month-old infants tended to select the ball that the experimenter previously had physical and verbal contact with, but not the unrelated one [12]. However, the cognitive components underlying the referent assignment process as well as its development are not well understood.

A previous study developed a “reference assignment task” that enabled evaluation in a natural conversational context of children’s response to ambiguous utterances that require anaphoric reference of the preceding explicit utterances [13] (see also the “cued recall” [14], “reading time” [5], and “priming technique” [15] measures for adult participants). In the task, children were shown cards presenting colorful objects (e.g., a yellow car, a red chair, etc.), and were required to answer four sets of five successive explicit and ambiguous questions. On the one hand, the explicit questions clearly required children to answer on one dimension of a presenting card (i.e., the name or color of the object on the card) with the utterance “What’s the name/color of this object?” On the other hand, semantic meaning of the ambiguous questions (i.e., “What about this?”) is arbitrary and thus identifying their referent required a pragmatic reference. Additionally, as an important manipulation, the task included a “topic change” of the explicit questions during the five successive questions’ sequence. For example, in a sequence starting with an explicit question regarding the name of the object, children were shown five different cards and asked: “What is the name of this?” “What about this?” “What color is this?” “What about this?” and “What about this?” (one question on one card). Besides testing the ability of tracking the semantics of explicit questions, the topic change raised a difficulty regarding answering the latter ambiguous questions (i.e., the second and third ones), because children needed to infer the intent of the ambiguous questions by retrospectively referring to the preceding explicit ones (in this case, the intent of the ambiguous questions was assumed to be consistent with the preceding explicit ones). It was shown that although 5-year-old children demonstrated an overall higher performance than 3-year-old children, they still misunderstood more ambiguous questions after the preceding explicit question had been changed to a different topic (i.e., the second and third ones) than the former ambiguous questions. These results suggested that the ability of tracking the explicit referential aspects develops during this period of development [13]. Further, with presenting the dissociation between the score of the referent assignment task and the dimensional change card sort task [16], Murakami and Hashiya [13] ruled out the possibility that the ability of cognitive shift could entirely explain the tendency to disambiguate a linguistic referent while retrospectively referring to the preceding contexts [13].

The current study aimed to further the knowledge regarding the basis of the pragmatic referential process by testing whether children’s performance on the referent assignment task is associated with phonological loop capacity. The phonological loop has been assumed to be a component of working memory; it is a hypothesized multicomponent system for temporarily storing and manipulating information [17–19]. Considering Baddeley’s three-component model of working memory, the phonological store might play a role as an “inner ear” where auditory verbal information enters automatically before being further processed. It consists of a phonological store, which can hold acoustic or speech-based information for 1 to 2 seconds, coupled with an articulatory control process [17], [18], [20–22]. Patients with a specific phonological loop deficit seem to have difficulty in comprehending certain types of complex sentences. Furthermore, the phonological loop may play a key role in vocabulary acquisition [23–24].

To correctly answer the questions in the referent assignment task, as mentioned above, detecting the intent of the questions while recalling the current/preceding explicit questions (as stored information) seems to be a prerequisite. Models related to the phonological loop for accounting how serial order is remembered propose that each phonological item might provide a cue for the next item (chaining models), or that successive items might be associated with an ongoing contextual cue (contextual models) [25–28]. Despite the different perspectives regarding these models, a hypothesis could be that a higher capacity on the phonological loop might result in an overall better performance on the referent assignment task (Hypothesis 1). That is, for the explicit questions, this capacity would help to remember the semantics of the current explicit question, and for the ambiguous questions, it would benefit in retrospectively recalling the information of the preceding explicit questions. This idea might also be supported by theories of inference processes, which assume that the concept to be inferred has to be brought into the working memory before further processing [15], [29].

However, one might argue that the capacity of the phonological loop might be only partially linked to the performance on the reference assignment task. That is, it might predict the performance on the explicit questions as well as the ambiguous questions before a topic change (i.e., the first ambiguous question), but not the ambiguous questions after the topic change (Hypothesis 2) [13]. That is, in the reference assignment task, children experience two explicit questions on different topics before answering the second and third ambiguous questions. This repeated-referent experience of explicit questions might interrupt the referential process by confusing children when accessing the expected antecedents for interpreting the intent of the ambiguous questions [30]. This pattern of results might rule out the effect of the phonological loop on disambiguating a linguistic referent and consequently encourage further possibilities regarding the integration of understanding the mechanism of properties of the referential process.

Specifically, the current study focused on 3–5 year old children who show a developing pragmatic referential ability in this period to extend the findings from previous studies [13]. Children’s capacity of the phonological loop was measured via a widely used test—the forward digit span test [31–33]. An experimenter read a series of single-digit numbers, and children were required to repeat them in the same order. The referent assignment task was conducted identical to that in the original studies [13]. In addition, we also examined children’s performance on the backward digit span test. It was conducted in the same way as the forward digit span test, except that the child was required to recall the digits in reverse order [18], [32], [34]. Although the storage component of this complex memory span task is also mediated by the phonological loop, its processing demands are supported by central executive resource. It has been suggested that this task is appropriate to be treated as reflecting the sub-ability of central executive, a hypothesized superior attention control system of the phonological loop in the working memory system [22]. Thus, we used this variable to mainly control the processing capacities of central executive to directly examine the effect of the phonological loop on referent assignment. Additionally, because increasing age might predict both the capacity of the phonological loop [22] and the performance on the reference assignment task [13], we post-analyzed the data by controlling for age by using generalized linear mixed models.

## Materials and methods

### Ethics statement

In accordance with the Declaration of Helsinki, the procedure was approved by the ethics committee of the Faculty of Human-Environment Studies at Kyushu University. All participants were native Chinese from middle-class backgrounds. They were recruited from (April 13–24, 2015) and tested in their kindergarten (May 11–14, 2015; Quancheng Garden kindergarten) in Jinan City in China. Local permission (written approval) from the principal of the kindergarten was received in order to conduct this research. For the participant recruitment, the children’s caregivers were informed about the procedure of the experiment including that the sessions will be recorded, and they gave their consent for their children’s participation.

### Participants and procedure

The final sample consisted of 76 children aged 3–5 years (43 girls; *M*_age_ = 50.1 months, *SD* = 8.04, *range* = 34 to 65.3 months). An additional nine children participated but were excluded from the analyses because they failed to answer the question regarding color (four children aged 39.2, 41, 41.2, and 49.2 months), did not show any response (one aged 34.2 months), cried (one aged 34.1 months), because of an experimental error as the experimenter forgot to conduct the digit span tasks or because of scheduling problems (three children aged 32.2, 34.1, and 54.1 months). Sample size was determined by the number of children that could be recruited.

The experiment was carried out in a quiet room in the kindergarten. Participants were tested individually by a male experimenter, while a female childminder was present to monitor the experiment. Each participant undertook two tasks, which are described below. All experimental sessions were video recorded (using a Panasonic HX-WA2, Japan).

### Referent assignment task

The referent assignment task was conducted identical to that in the previous study [13].

#### Stimuli

**Fig 1:**
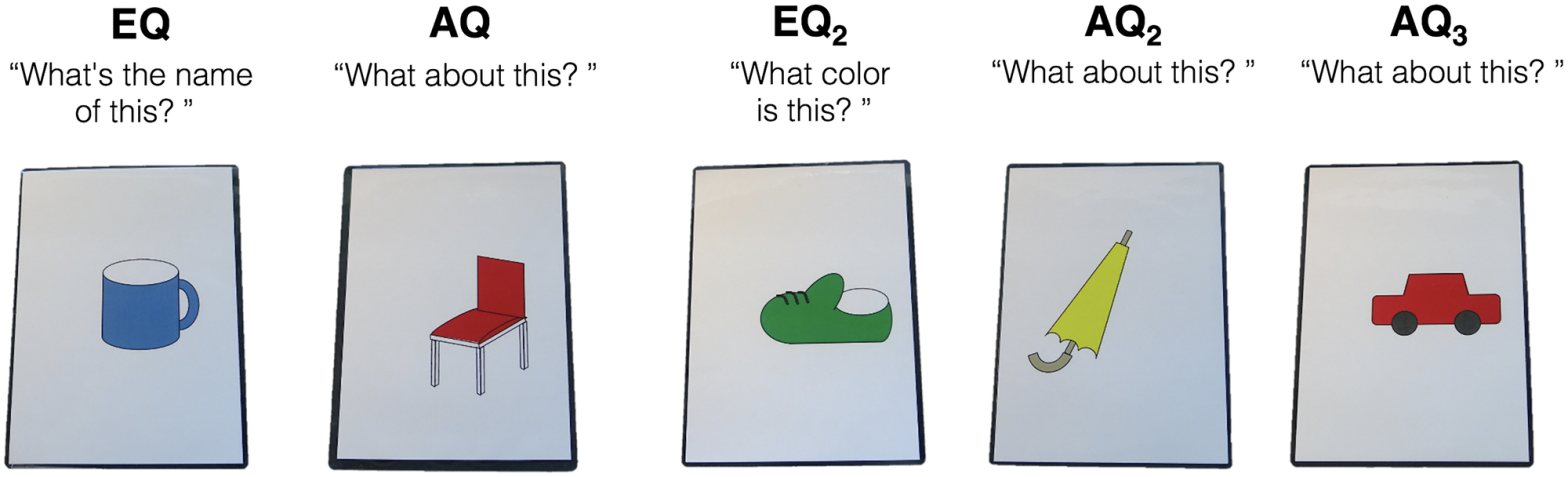
Sample of the laminated cards used in a trial in the reference assignment task. The five questions were in a fixed order of EQ/AQ/EQ_2_/AQ_2_/AQ_3_.

The experimenter asked participants questions regarding illustrations on laminated cards (14.8 × 21 cm), while showing the cards as a reference. Each card represented one of five kinds of illustrations (i.e., umbrella, shoe, chair, cup, or car) in one of four colors (i.e., red, blue, yellow, or green) (Fig 1).

#### Procedure

The task included four trials. Each trial consisted of five questions in turn (i.e., twenty questions in total); each was an explicit question (EQ; i.e., “What's (the name of) this?” [in Chinese “这是什么东西?”], or “What color is this?” [in Chinese “这是什么颜色?”]), or an ambiguous question (AQ; e.g., “What about this?” [in Chinese “这个呢?”]) about the illustration. The sequence of questions included in a trial was fixed as EQ, AQ, EQ_2_, AQ_2_, AQ_3_. The EQ and EQ_2_ in a sequence concerned different dimensions (name/color) of the illustration. Two trials of the task began with the EQ regarding the name of the object, which presented a NWCWW (NC) sequence: N (*name*; i.e., “What's the name of this?”), W (*what*; i.e., “What about this?”), C (*color*; i.e., “What color is this?”), W (*what*; i.e., “What about this?”), W (*what*; i.e., “What about this?”). The other two trials began with the EQ regarding color, presenting a CWNWW (CN; *color*, *what*, *name*, *what*, *what*) sequence. The order of the trials including the different types of sequences was counterbalanced across participants.

The experimenter showed the cards and said to the children (in Chinese), “I will show you some of these cards and ask you some questions. Listen to me carefully and answer the questions.” The experimenter then conducted the task. The experimenter made eye contact with the children, and responded rather positively by nodding in response to the child’s answer, regardless of whether it was correct or not. After one trial, the experimenter recorded the answers by filling in an answer sheet, and aligned the five cards in front of the child to indicate the completion of that trial, and then took out five new cards for the next trial.

#### Scoring

The answers were coded as 1 (correct) or 0 (incorrect). Note that because this task was used to investigate a child’s response to a question when its referent (i.e., the name or the color) was clear (in EQ and EQ_2_) and ambiguous (in AQ, AQ_2_ and AQ_3_), an answer that referred to the identical dimension that the question intended to indicate was treated as correct. That is, for instance, the answer of “blue” is correct when a child is shown a red car card and asked “What color is this?” For the AQ, AQ_2_ and AQ_3_, the answer that referred to the dimension that the preceding explicit question (i.e., the EQ or EQ_1_) referred to was treated as correct.

Additionally, to ensure that the current investigation on the relationship between children’s phonological loop and referent assignment contributes to extending the findings from previous studies [13], we also applied the following coding battery that presents the sequential pattern of a child's response according to the previous study.

The Base-Assignment Score reflected whether the child had appropriately identified the reference in the absence of a topic shift. It was coded as 1 when both the EQ and AQ were correct.

The Shift Score indicated whether the child’s response changed with the switching of the referent according to the explicit questions. It was coded as 1 when both the EQ and EQ_2_ were correct.

The Re-Assignment Score denoted a child's referential assignment based on the topic shift. It was coded as 1 when both the EQ_2_ and AQ_2_ were correct.

The Follow-Re-Assignment (Follow-RA) Score indicated whether the child interpreted the repetition of the same ambiguous question consistently. It was coded as 1 when both the AQ_2_ and AQ_3_ were correct.

### Digit span tasks

All participants were administered the forward and backward digit span task [31–33]. The experimenter verbally presented digits at a rate of one per second. The participant was required to repeat the digits verbatim or in reverse order, in the forward or backward test, respectively. The number of digits increased by one until the participant consecutively failed two trials of the same digit span length. Then the number of digits that preceded the consecutive failures was treated as the score. No feedback was given to the child throughout the task. After each test, the experimenter recorded the answers by filling in an answer sheet. We termed the scores of the forward digit span test as Phonological Loop Scores (PLSs), and the scores of the backward digit span test as Central Executive Scores (CESs).

### Data analysis

Statistical analyses were conducted using R, version 3.2.4 (R Foundation for Statistical Computing, Vienna, Austria). All reported p values are two-tailed.

First, we investigated children’s performance on the reference assignment task. We used a nonparametric analysis of variance equivalent (Friedman rank sum test) [35], to determine if at least two of these five answer scores were significantly different from each other. The Wilcoxon signed-rank test was then used to compare each of these five with each other; p values were adjusted using Bonferroni adjustment [36].

Second, to test the effect of PLSs (range = 0–8) on the response variable (i.e., children’s scores on the referent assignment task; 1 or 0), we applied linear mixed models (GLMMs) with binomial error function and logit link function to analyze the overall dataset. The models also included CES (range = 0–2), age, pattern (CN or NC), and sex (male or female) as fixed variables and individual differences as a random effect for the following reasons.

First, consistent with the results of previous studies [13], [22], [37], our preliminary analysis revealed significant correlations between PLS and age (Spearman’s ρ = 0.481, p < .001), PLS and CES (ρ = 0.3, p < .001), and CES and age. (ρ = 0.587, p < .001). That is, the CES and age might be confounding variables that increase the bias of the estimated effect of PLS on the children’s responses due to the presence of a common cause [38]. After confirming the absence of multicollinearity of the predictors by using the Variance Inflation Factor (VIF) (VIF_PLS_ = 1.32, VIF_CES_ = 1.42, VIF_age_ = 1.73; using the VIF function in R) [39], we used full models to directly investigate the effects of PLS (i.e., adjusted effects) while controlling CES and age as explanatory variables. Second, Murakami and Hashiya [13] reported that 5-year-olds tended to answer with the name of the object in response to an ambiguous question (i.e., AQ, AQ_2_ and AQ_3_) [13]. Therefore, we included the type of the order of questions as an explanatory variable (i.e., the pattern factor; CN or NC) to confirm the tendency. Third, although no prediction was established regarding the effect of sex on the children’s performance, considering that the current study was conducted by a male experimenter, we included sex as an explanatory variable to confirm that female and male children did not respond differently due to this specificity. Fourth, because we had repeated measures (four trials) from the same participants, we treated individual differences as random effects in the models. In addition, we also tested simpler models to confirm the significant effects by dropping single terms and applying likelihood ratio tests (LRTs; using the lrtest function in R).

## Results

### Performance on the referent assignment task

Descriptively, children showed a decreasing performance on the five answers of the referent assignment task. Table 1 showed the mean scores and standard deviations for each answer, and the mean differences between each of the answers (Table 1). Considering that a significant difference among the five answer scores was indicated (Friedman χ^2^ (4) = 52.319, p < .001), post-hoc multiple comparison tests showed that children performed better on the EQ, AQ, and EQ_2_, than on the AQ_2_ and AQ_3_ (Wilcoxon signed-rank test; adj.ps < .01).

**Table 1:** Children's performance on the tasks.

Questions and Tasks	Mean (SD)	EQ	AQ	EQ2	AQ2	AQ3
**EQ**	0.91 (0.283)	-	0.017	0.034	0.128***	0.145***
**AQ**	0.90 (0.306)		-	0.017	0.111***	0.128***
**EQ2**	0.88 (0.327)			-	0.095***	0.111***
**AQ2**	0.78 (0.412)				-	0.017
**AQ3**	0.77 (0.423)					-
**forward digit span test**	4.80 (1.516)					
**backward digit span test**	0.51 (0.721)					

Mean score and standard deviation (in parentheses) for each answer and task, and the difference between means of each two answers of the reference assignment task were demonstrated.

***adj. p < 0.01.

Furthermore, although the experimenter’s clear demonstration of the completion of the first trial and the beginning of the second trial seemed to avoid a carry-over effect (i.e., effects of experiencing the first trial on the answers in the second trial), to confirm this, we compared the AQ scores in the two paired trials. That is, if children have treated AQ in the second trial as an “AQ_4_,” their performance on that question would be expected to be worse than that on the AQ in the first trial, as well as their performance on AQ_2_ or AQ_3_ in the first trial. However, the results showed that there was no difference in the performance between the first and second AQ (Wilcoxon signed-rank test; p = .132).

### Relationships between phonological loop scores and performance on the referent assignment task

**Fig 2:**
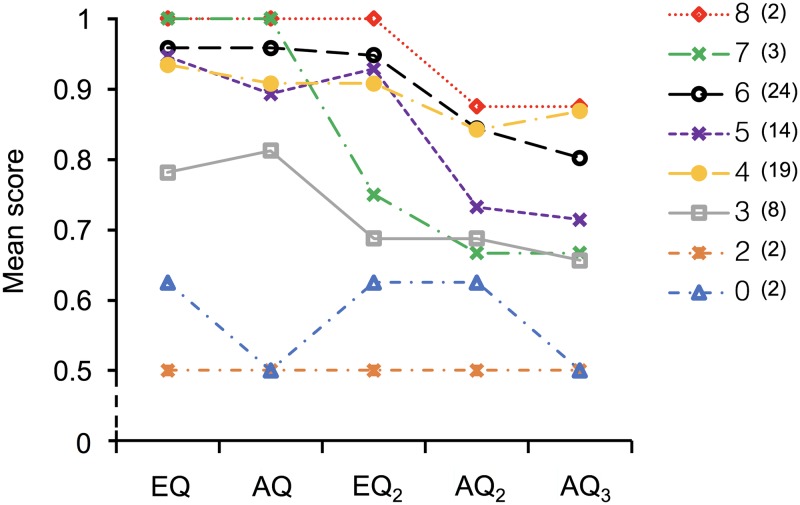
Line chart represents the mean scores of each question of the referent assignment task in children with different PLS scores. Colored lines represent performance of groups of participants with different PLS scores ranging from 0–8 (i.e., 0, 2, 3, 4, 5, 6, 7, 8) in the current study. Numbers of participants are shown in the parentheses.

The performance on each question of the referent assignment task of children with different PLS scores is shown in Fig 2. We used the full models to test the effects of PLS on children’s performance on the referent assignment task while controlling the effects of potential confounders. The results showed that the PLS had significant positive effects on the EQ score (*β* = 0.409, *z* = 2.85, p = .004; LRT; *χ*^*2*^ = 8.24, *df* = 6, p = .004), the AQ score (*β* = 0.414, *z* = 2.99, p = .003; LRT; *χ*^*2*^ = 9.4, *df* = 6, p = .002) and the EQ_2_ score (*β* = 0.259, *z* = 2.05, p = .041; LRT; *χ*^*2*^ = 4.12, *df* = 6, p = .042). However, this was not the case for the AQ_2_ score (*β* = 0.098, *z* = 0.84, p = .399; LRT; *χ*^*2*^ = 0.71, *df* = 6, p = .4) or the AQ_3_ score (*β* = 0.103, *z* = 0.88, p = .38; LRT; *χ*^*2*^ = 0.76, *df* = 6, *p* = .382) (Table 2).

**Table 2:** GLMM analysis for predicting reference assignment task scores.

Model	Fixed effects					Random effects	Deviance -2log L*
	PLS	CES	age	type (1 = CN, 0 = NC)	sex (1 = male, 0 = female)		
1	0.409*** (0.144)	1.126 (0.714)	0.040 (0.036)	-0.193 (0.440)	-0.436 (0.445)	Participants trials	145.2
2	0.414*** (0.138)	1.026 (0.596)	0.034 (0.034)	-0.764 (0.424)	-0.362 (0.416)		162.8
3	0.259* (0.127)	0.724 (0.474)	0.050 (0.031)	-0.001 (0.374)	-0.198 (0.380)		192.6
4	0.098 (0.116)	0.270 (0.282)	0.017 (0.026)	0.513 (0.296)	-0.034 (0.323)		299.2
5	0.103 (0.117)	0.182 (0.276)	0.017 (0.026)	0.699* (0.293)	-0.010 (0.324)		309.8
						Observations 296	
						Subject 74	

Models 1–5 represent the GLMMs predicting EQ, AQ, EQ2, AQ2, AQ3 as response variables, respectively (scores = 1 or 0). All models included PLS, CES, age, type, and sex as fixed variables and individual differences as a random effect. Standard errors shown in parentheses.

***p < 0.01,

*p < 0.05.

Moreover, type had a significant effect on the AQ_3_ score (*β* = 0.699, *z* = 2.39, p = .017; LRT; *χ*^*2*^ = 5.87, *df* = 6, p = .015). Participants showed higher scores in this question in which the preceding explicit question (EQ_2_) was the name rather than the color dimension. In other words, when asked with an NC (i.e., NWCWW) sequence, in which the topic of explicit questions was changed from the name to the color dimension, children tended to answer the AQ_3_ by referring “erroneously” to the name of the objects. In addition, results did not reveal a significant effect of sex on children’s performance on the referent assignment task (ps > .32).

The similar results were observed when we used the coding battery according to the previous study [13] (Table 3). The PLS had significant positive effects on the Base-Assignment Score (*β* = 0.450, *z* = 3.29, p < .001; LRT; *χ*^*2*^ = 11.06, *df* = 6, p < .001) and the Shift Score (*β* = 0.487, *z* = 2.52, p = .012; LRT; *χ*^*2*^ = 6.75, *df* = 6, p = .009). However, this was not the case on the Re-Assignment Score (*β* = 0.18, *z* = 1.56, p = .118; LRT; *χ*^*2*^ = 2.44, *df* = 6, p = .118) or the Follow-Re-Assignment Score (*β* = 0.114, *z* = 0.93, p = .353; LRT; *χ*^*2*^ = 0.86, *df* = 6, *p* = .353). Moreover, type had a significant effect on the Follow-Re-Assignment Score (*β* = 0.639, *z* = 2.22, p = .027; LRT; *χ*^*2*^ = 5.06, *df* = 6, *p* = .024). Results did not reveal a significant effect of sex on children’s performance on the referent assignment task (ps > .26).

**Table 3:** GLMM analysis for predicting referent assignment task scores using the coding battery (Murakami & Hashiya, 2014).

Model	Fixed effects					Random effects	Deviance -2log L*
	PLS	CES	Age	type (1 = CN, 0 = NC)	sex (1 = male, 0 = female)		
1	0.450*** (0.137)	1.125 (0.594)	0.030 (0.032)	-0.637 (0.407)	-0.448 (0.403)	Participants trials	170.2
2	0.487* (0.193)	0.916 (0.521)	0.071 (0.043)	-0.134 (0.366)	-0.334 (0.533)		229.0
3	0.180 (0.115)	0.277 (0.283)	0.023 (0.026)	0.457 (0.291)	-0.093 (0.320)		305.8
4	0.114 (0.123)	0.140 (0.286)	0.024 (0.027)	0.639* (0.289)	-0.003 (0.338)		323.4
						Observations 296	
						Subjects 74	

Models 1–4 represent the GLMMs predicting Base Assignment Score, Shift Score, Re-Assignment Score, Follow-Re-Assignment Score as response variables, respectively (scores = 1 or 0). All models included PLS, CES, age, type, and sex as fixed variables and individual differences as a random effect. Standard errors shown in parentheses.

***p < 0.01,

*p < 0.05.

## Discussion

The current study aimed to further the knowledge of the background of the developing pragmatic referential capacity in children by investigating whether the phonological loop affected referent assignment. On the one hand, children were administered the referent assignment task in which an experimenter asked them to answer five successive questions that included explicit and ambiguous ones in the following order: EQ, AQ, EQ_2_, AQ_2_, and AQ_3_. The explicit questions clearly asked about either the name or the color of a colorful illustration of an object, whereas the ambiguous questions (i.e., “What about this?”) required an anaphoric reference of the preceding explicit utterances. Additionally, there was a topic shift between the first and second EQs. On the other hand, the phonological loop capacity was measured by using the forward digit span task in which children were required to repeat the numbers as an experimenter uttered them. The results from the GLMMs predicting children’s performance on the referent assignment task showed that the scores on the forward digit span task had a positive effect on the correct response on the explicit questions as well as the ambiguous questions before the topic shift, but not the ambiguous questions after the topic shift. Moreover, children showed a better performance on the last ambiguous question, in which the current topic of the explicit question had been changed from the color to the name dimension, compared to the inverse situation. Additionally, although the current study was conducted by a male experimenter, female and male children did not respond differently on the referent assignment task depending on the experimenter’s gender.

To give an appropriate response in the referent assignment task, children needed to correctly detect the referent of the explicit questions while tracking the semantics, and the referent of the ambiguous questions while retrospectively referring to the preceding explicit questions. Previous research on working memory has demonstrated that the phonological loop, including a phonological store and articulatory control process, contributes to the storage of auditory verbal information [17], [18], [31–32]. This might predict that the phonological loop could positively affect the response on both the explicit and ambiguous questions while benefiting preservation of the semantic information of the explicit questions. However, the predictions were partially supported (as predicted in Hypothesis 2). Since that the context before the topic shift may have limited the interpretation of the first ambiguous question to be processed in accordance with the first explicit question, this ambiguous question is thus in fact contextually explicit. In this sense, the current results suggested that the interpretation process of questions that are explicit in semantics or pragmatics is modulated by the phonological loop. However, children’s referential process involved in the latter ambiguous questions after experiencing multiple topics in the explicit questions seems to require other cognitive components. Children performed worse on those questions than the first ambiguous question (as shown in Table 1) and most importantly, the phonological loop seems to be irrelevant in this backward referential process (as shown in Table 2) [30]. Note that our predictions presumed a linear relationship between phonological loop capacity and referent assignment. Therefore, the current results could also be used to rule out a negative correlation between them, that is, the possibility of a better phonological short-term memory regarding the first explicit question, which is supported by a higher phonological loop capacity, might have a negative effect on the performance on the latter ambiguous questions (i.e., good short-term memory increase the tendency of answering the latter ambiguous questions in accordance with the first explicit question), and vice versa. However, the current presumption could not test any nonlinear relationships (e.g., U-shaped or inverted-U-shaped) between phonological loop capacity and referent assignment, although the performance on the latter two ambiguous questions seems to be independent from the phonological loop capacity (Fig 2).

The dissociation between the phonological loop and the response to the latter ambiguous questions also seems to suggest that the disambiguation might not accompany a retrospective reference process on the preceding information of the explicit questions. Based on the referential theory [40–41], previous studies of structural ambiguity resolution have proposed that the discourse might be consulted only in the face of ambiguity (the ambiguity only hypothesis), or that the referential support emerges from fundamental properties of how we access and update mental models of discourse (the interaction models) [42–43]. That is, if a backward referential process only works when children perceive the ambiguity of the question “What about this?” (as predicted in the ambiguity only hypothesis), children might retrospectively retrieve the information of the preceding explicit question, which might be strongly supported by the phonological loop capacity, as discussed above. However, this link was not observed. Future research should investigate whether the referential process updates along with the progression of the discourse.

The effects of phonological loop capacity on the referent assignment task did not change when we used a coding battery that presents the sequential pattern of a child's response according to the previous study, in which the children’s ability of cognitive shift (measured by the dimensional change card sort task) appeared to be associated with correctly answering an explicit question, but not disambiguating a linguistic referent while retrospectively referring to the preceding contexts [13]. Together, these studies might reduce the possibilities that the pragmatic referential process could be directly supported by cognitive shift and working memory, which have been described as a part of executive function [44–46]. At the same time, they might further raise questions regarding the understanding of the underlying mechanism of the referential process.

It is also interesting that children performed better on the last ambiguous question after the topic shift in which the topic of the preceding explicit question had been changed to the name dimension, compared to the inverse situation. That is, children seemed to be more likely to answer with the name of the object rather than the color in this ambiguous question. The previous study revealed this tendency in 5-year-olds, but not 3-year-old children and proposed that it might be due to the bias which could be confirmed in 2-year-olds that certain dimensions of perceptual similarity (i.e., shape) are weighted more heavily than others in determining word extension [13], [47]. Consistent with these findings, the current results using a broader participant age range, might demonstrate that this “shape bias” seems to be observed in an earlier developmental stage.

The possibility of cultural differences in children's communication on the basis of pragmatic referential process should be another focus of future research. We tested Chinese children in the current study. After controlling for PLS and CES, we found that age seems to be unrelated to the response to explicit and ambiguous questions. However, the developing performance on the reference assignment task in 3- to 5-year-old children was revealed in the previous study (in Japanese children [13]), as well as the models without the PLS and CES as fixed factors (in pilot analysis of the current study). This evidence might imply the existence of other related abilities of the referential process that might be correlated to age, and the possible parallel developmental roots in children with different cultural backgrounds. Future researches might investigate these questions by, for example, directly comparing children’s performance on the reference assignment task with using identical measures, or comparing their developmental strategies with taking into account the cultural differences in the ToM [48–50] and social attention [51– 52].

Several potential limitations of the current study need to be considered. First, since phonological loop contributes to language learning, it is possible that the verbal intelligence plays a modulator role in the observed relationships between phonological loop capacity and referent assignment [24], [53]. Although we had excluded the data of children who did not understand the explicit questions from our analysis, and the unpublished data on children with autism spectrum disorder showed that the verbal comprehension (assessed by Japanese version of Wechsler Intelligence Scale for Children) does not directly relate to the performance of referent assignment task (Murakami, in preparation), the possibility needs to be directly tested in further investigations. Second, the referent assignment task in fact involves set shifting between answering the “color” and “shape.” Murakami and Hashiya [13] demonstrated that the performance on the referent assignment task in both 3- and 5-year-old children was not influenced by whether they could pass a dimensional change card sort task which reflects the cognitive flexibility [13], and the current results showed that children answered the second explicit question correctly as the first one, suggesting their success in the explicit set-shifting. However, neural evidence has indicated that the activity in dorsolateral prefrontal cortex, which seems to play an important role as a putative control structure, shows a strong relationship with successful performance on rule shifting task [54]. Therefore, it remains possible that the set-shifting ability has modulated the relationship between the phonological loop capacity and referent assignment. Together, further research is needed to investigate the underlying cognitive components of referent assignment using more constructive designs including assessments that cover a wide range of possibly related abilities (e.g., verbal intelligence and set-shifting).

The disambiguation of communicative expressions on the basis of tracking the contextual information is essential for communication and social interactions [1], [3]. Although the current data failed to elicit any hypothesized linear relationship between phonological loop capacity and the interpretation of the referent of an ambiguous utterance, these results are expected to contribute to the understanding of the underlying mechanism of pragmatic referential abilities in children, while distinguishing the backgrounds of the responses toward explicit and ambiguous utterances. Taken together with the previous findings that performance on the referent assignment task is irrelevant to cognitive shift [13], it seems that further research on pragmatic referential development requires perspectives that go beyond executive function to other mental processes.

## Supporting information

S1 Dataset (XLSX)
